# Biogeographic Insights Into the Late Miocene Diversification of the Giant Deep‐Ocean Amphipod *Eurythenes*

**DOI:** 10.1002/ece3.70730

**Published:** 2025-01-23

**Authors:** Carolina E. González, Johanna N. J. Weston, Reinaldo Rivera, Marcelo Oliva, Rubén Escribano, Osvaldo Ulloa

**Affiliations:** ^1^ Instituto Milenio de Oceanografía (IMO) Universidad de Concepción Concepción Chile; ^2^ Biology Department Woods Hole Oceanographic Institution Woods Hole Massachusetts USA; ^3^ Instituto de Ciencias Naturales Alexander von Humboldt, Facultad de Ciencias del Mar y Recursos Biológicos Universidad de Antofagasta Antofagasta Chile; ^4^ Departamento de Oceanografía Universidad de Concepción Concepción Chile

**Keywords:** abyssal, BioGeoBEARS, global change biology, hadal, historical biogeography, mitochondrial DNA, time‐calibrated phylogeny

## Abstract

Mechanisms driving the spatial and temporal patterns of species distribution in the Earth's largest habitat, the deep ocean, remain largely enigmatic. The late Miocene to the Pliocene (~23–2.58 Ma) is a period that was marked by significant geological, climatic, and oceanographic changes. This transitional period spurred widespread species diversification, particularly among widely distributed benthic scavengers, such as amphipods. Here, we take step toward understanding the long‐term evolutionary processes of amphipod colonization and diversification in the deep ocean by focusing on the model genus *Eurythenes* S. I. Smith in Scudder, 1882. These large‐bodied scavengers play key roles in benthic communities. We constructed a time‐calibrated phylogeny using two mitochondrial DNA genes by analyzing publicly available data on 14 species of *Eurythenes* across a global depth range from 839 to 8081 m. The resulting phylogenetic tree reveals a diverse clade, with a common ancestor originating around 11.81 Ma. A gradual increase in the effective population size of *Eurythenes* was observed, particularly during the Pliocene (~4 Ma). The net diversification rate remained almost constant, with slight increases between the Miocene and Pliocene (~8–4 Ma), and most new species appeared during the latter period. Additionally, reconstruction of the ancestral area suggested that the common ancestor of *Eurythenes* had a global distribution. A combination of dispersal and sympatric processes, along with environmental factors, such as changes in ocean temperature and sea level, contributed to the present biogeographic distribution of these species. Our findings highlight the importance of historical events, such as plate tectonics and changes in deep‐water circulation, in driving the rapid speciation of *Eurythenes* and underscore their essential role in shaping deep‐ocean biodiversity.

## 1 Introduction

Understanding the historical factors that have triggered evolutionary radiation or large‐scale extinctions can be addressed by identifying species‐rich taxonomic groups with global distributions and high diversity. These evolutionary processes, coupled with global environmental changes and dispersal, have modulated species richness at different scales (Roelants et al. 2007; Hannisdal and Peters 2011). The deep ocean, which extends beyond 200 m, provides a suitable model for investigating how evolutionary processes may interact with large‐scale environmental events or processes on a global scale. Given that the deep ocean encompasses 66% of the seafloor (Rex et al. 1993; Woolley et al. 2016), it has traditionally been thought of as having limited potential for driving speciation, with few barriers promoting reproductive isolation (Palumbi 1994; Bierne, Bonhomme, and David 2003).

The organization of taxa is observable across various spatial scales, ranging from marine basins (Weston et al. 2022) to isolated habitats, such as whale falls (Smith et al. 2015) and hydrothermal vents (Lonsdale 1977). Moreover, the deep ocean is a rich source of biodiversity, contrary to the historical belief that deep‐ocean diversity is low owing to extreme environmental conditions (Hessler and Sanders 1967). Studies have documented substantial species diversity in deep‐sea ecosystems (e.g., polychaetes, bivalves, and foraminifera; Sanders 1968; Allen and Sanders 1996; Olabarria 2005), particularly in organisms with limited dispersal capabilities, which more accurately reflect the historical processes that influenced their evolution in response to significant changes in physical and chemical variables during geological history (Zachos et al. 2001; Yasuhara et al. 2008; Sweetman et al. 2017). A possible explanation is that tectonic changes and other oceanographic factors may create effective physical barriers to dispersal, leading to vicariance events and allowing allopatric or parapatric speciation (Palumbi 1994). However, in the marine environment, sympatric speciation is quite common (Bowen et al. 2013), and this typically occurs when factors such as natural selection, sexual selection, or adaptation to different ecological niches within the same area lead to genetic and reproductive divergence (Munday, Van Herwerden, and Dudgeon 2004). Nonetheless, it is unclear which types of speciation predominate in the deep ocean and what factors have driven the evolution and proliferation of organisms, such as crustaceans, in this apparently inhospitable environment.

Among crustaceans, Amphipoda are one of the most morphologically and ecologically diverse orders, inhabiting all aquatic environments from alpine streams to the deepest point in the ocean, making them an intriguing taxon for gaining insight into historical biogeographic processes (Barnard and Karaman 1987). As peracarids, amphipods have a limited dispersal capacity owing to their brooding reproduction, absence of free‐swimming larvae, and extended parental care. As such, populations are more susceptible to extinction or more capable of speciating across geographical barriers due to the cessation of gene flow (Barnard and Karaman 1987; Thiel 1999; Väinölä et al. 2008; O'Donovan, Meade, and Venditti 2018; Avaria‐Llautureo et al. 2021). With their global distribution, high abundance, and ecological significance, amphipods provide a system for understanding the impacts of current and historical events.

In the deep ocean, *Eurythenes* S. I. Smith in Scudder, 1882 is a model genus for studying historical biogeography, in addition to ecology, physiology, and reproduction (Hargrave 1985; Ingram and Hessler 1987; Christiansen and Pfannkuche 1990; Thurston, Petrillo, and Della Croce 2002; Blankenship et al. 2006; Premke, Klages, and Arntz 2006; Blankenship and Levin 2007; Premke and Graeve 2009; Reid, Cuomo, and Jamieson 2018). *Eurythenes* are found from bathyal depths (~500 m) to hadal depths (~8000 m) across all oceans, from the poles to the tropics. They have become iconic as specimens are large (up to ~14 cm), highly mobile, and can be relatively easily collected with several methods, including baited traps and midwater trawls. *Eurythenes* play a crucial role in benthic communities as benthopelagic scavengers, apart from *Eurythenes obseus* being only pelagic. Functioning like vultures, *Eurythenes* efficiently consume and recycle carbon originating from the surface (Desbruyères 1985; d'Udekem d'Acoz and Havermans 2015). *Eurythenes* are strong swimmers in the water column, as emphasized by their rapid arrival to bait in swarms and a collection record of individuals 1800 m above the bottom (Baldwin and Smith 1987). They are also considered long‐living (~10 years), which suggests that an individual has the potential for a wide geographic and bathymetric range.

At first glance, the biogeography of *Eurythenes* as a globally distributed genus appears straightforward. However, the picture has become highly complex with the recent discovery of cryptic speciation. Since 2015, *Eurythenes* has expanded from five to ten named species and several undescribed species (d'Udekem d'Acoz and Havermans 2015; Narahara‐Nakano, Nakano, and Tomikawa 2018; Weston et al. 2020, 2021). The improved identifications revealed *Eurythenes'* bathymetric and geographic distribution to be highly idiosyncratic and include instances of sympatry. Some species, like *Eurythenes magellanicus* and *Eurythenes maldoror*, have a cosmopolitan distribution across abyssal depths. Once considered cosmopolitan, *Eurythenes gryllus* is now presumed amphitropical to the Arctic and Southern oceans. Additionally, certain species appear restricted to one basin, like *Eurythenes andeakarae* in the Southern and South Atlantic oceans. Several species are even endemic, such as *Eurythenes atacamensis*, which is only present in the abyssal to hadal depths of the Peru‐Chile Trench. The combination of diverse distributions and biological factors suggesting high dispersal ability leaves many unresolved questions about how historical factors shaped present‐day distributional patterns.

While *Eurythenes* is commonly used as a model taxon for deep‐ocean benthos, there is limited knowledge of their spatial distribution. Initial studies suggest that the *Eurythenes* lineages emerged around 10–8 Ma (Corrigan et al. 2014; Copilaş‐Ciocianu, Borko, and Fišer 2020), but these only included two of the ten species and lacked sufficient resolution to comprehensively address the rise and global diversification across the bathyal to hadal seafloor. During the late Miocene to early Pliocene (∼9–3.5 Ma), significant geological, climatic, and oceanographic changes likely played a role in the widespread diversification of species (Haywood et al. 2008; Steinthorsdottir et al. 2021). For example, the closure of the Isthmus of Panama (∼7.0–3.6 Ma), uplift of the Andes (~10–6 Ma), isolation of the Mediterranean Sea (∼5–6 Ma), expansion of Antarctic ice (∼14 Ma—17 kyr), and changes in the deep circulation pattern (9–3.5 Ma) (Hodell and Kennett 1985; Prell and Kutzbach 1992; Molnar, England, and Martinod 1993; Quade and Cerling 1995; Cerling et al. 1997; Zhisheng et al. 2001; Zachos et al. 2001; Herbert et al. 2016; Holbourn et al. 2018; Westerhold et al. 2020; Steinthorsdottir et al. 2021) were all large‐scale events that may have influenced global cooling. These events then lead to reduced vertical thermal gradients and a deeper thermocline, reducing zonal and meridional gradients globally (Brierley and Fedorov 2010). These large‐scale events had variable impacts on marine organisms, resulting in an increase in diversification for some species, such as diatoms and planktonic and benthic foraminifera (Smart, Thomas, and Ramsay 2007; Lewitus et al. 2018; Wade et al. 2018), while other organisms, such as bivalves, gastropods, and dinoflagellates (Taylor, Hoppenrath, and Saldarriaga 2008; Kiel and Nielsen 2010) experienced a decline in diversity. The precise impact of these changes on the speciation of *Eurythenes* remains unclear, especially regarding how they influenced their colonization over the seafloor.

In this study, we focused on providing stronger estimates on the timing of diversification to gain a deeper understanding of the historical biogeography of *Eurythenes*. Using publicly available data, we developed a time‐calibrated molecular phylogeny of the genus globally to establish species relationships and determine the temporal lineages of species divergence. We then reconstructed the ancestral geographic area of the genus to understand the potential consequences of the late Miocene to Pleistocene environmental changes on the global proliferation of this taxonomic group across the deep ocean. Testing the factors that shaped *Eurythenes*' present distribution provides a window into future biogeographic distribution for this genus and other deep‐benthos invertebrates with a perspective of the future deep‐ocean environment under ongoing global climate change and anthropogenic impacts.

## 2 Materials and Methods

### 2.1 Data Collation: Occurrence Records and Genetic Sequences

We compiled publicly available occurrence records and DNA sequences for *Eurythenes*. The occurrence records were obtained from the Ocean Biodiversity Information System (OBIS), Global Biodiversity Information Facility (GBIF), and specialized literature. These online databases were accessed on June 13, 2024, using the “robis” and “rgbif” packages in R v4.3 (Whiting et al. 2016; Chamberlain, Oldoni, and Waller 2017; Team Rs 2020). Records lacking geographic information, with coordinates equal to zero, or located on continents were excluded. Only species‐level records were selected, and duplicate records were excluded (see Data S1).

The genetic dataset was divided into available partial sequences from the small subunit ribosomal RNA (*16S*) and cytochrome oxidase I (*COI*) genes for 14 species of the genus *Eurythenes* (see Table S1 in Data S2), comprising named species or undescribed but distinct lineages. Most sequences were publicly available and obtained from GenBank.

An additional *E. atacamensis* individual was added to the genetic dataset following the ATACAMA HADAL expedition in the austral summer of 2022. The individual was collected using baited traps attached to a lander at a depth of 6500 m. Genomic DNA was extracted from pleopods 1 and 2 using the Mollusca kit (Omega), strictly following the manufacturer's protocol, except for incubation with lysis buffer and proteinase K for 3 h (González et al. 2020). Amplification of *16S* and *COI* was performed following the protocol described by Weston et al. (2021). The primer sets used for amplification were AMPH1 (France and Kocher 1996) and ‘Drosophila‐type’ 16SBr for *16S* (Palumbi 2002) and LCO1490 and HCO12198 (Folmer et al. 1994) for *COI*. The PCR products were sequenced by the sequencing service of Macrogen Inc. (Seoul, South Korea). Consensus sequences from both directions for *E. atacamensis* were constructed using Geneious Prime 2024.07 (Duran et al. 2012) and compared using BLASTn to verify the absence of contamination. The *COI* sequences were translated to amino acids to confirm the absence of unexpected termination codons.

The alignments for each gene fragment were created to estimate phylogenetic relationships and diversification. The final alignments for each gene fragment were performed using MUSCLE (Edgar 2004) and Geneious Prime 2024.07 (Duran et al. 2012). Fourteen *Eurythenes* sequences were aligned for *16S* and 11 for *COI*. For outgroups, *Cyclocaris* sp. Chevreux, 1899 (Family: Cyclocaridae) was selected as the closed‐related taxon in the shared superfamily, and *Alicella gigantea* Chevreux, 1899 (Family: Alicellidae) as from a separate superfamily [*Cyclocaris* sp. GenBank accessions: KF430272 (*16S*) and KP713899 (*COI*); *Alicella gigantea* : KP456083 (*16S*) and KP713893 (*COI*)]. The resulting alignment lengths for *16S* and *COI* were 295 and 660 bp, respectively. The alignments were concatenated and analyzed together in all subsequent steps.

### 2.2 Phylogenetic Analyses and Diversification Timing

Phylogenetic analyses were conducted using. Maximum Likelihood (ML), likelihood‐ratio test(SH‐aLRT) (Guindon et al. 2010) and Bayesian Inference (BI) methods. The ML and SH‐aLRT analyses were conducted using partitioned data in IQ‐TREE v2.2 (Minh et al. 2020). For the SH‐aLRT analysis, the MFP + MERGE function was used to determine the optimal partition scheme and substitution models, while the TEST function was applied for the ML analysis. Support values were estimated using 5000 bootstrap replicates for the ML analysis and 5 × 10^6^ replicates for SH‐aLRT. The BI analysis co‐estimated the trees and divergence times and was conducted using BEAST v2.7. (Bouckaert et al. 2014). The evolutionary model for each gene was determined using the bModelTest (Bouckaert and Drummond 2017), employing an optimized relaxed clock adjusted for each gene under a birth‐death model with exponential distribution. The average clock substitution rate for the *16S* gene was set at 0.0054 substitutions per million years and for the *COI* gene at 0.0177 substitutions per million years, which are standard for arthropods (Papadopoulou, Anastasiou, and Vogler 2010). Four simultaneous Markov chain Monte Carlo (MCMC) runs were executed for 5 × 10^8^ iterations, sampling every 1000 generations. All estimated parameters had effective sample size (ESS) values exceeding 200 and were visualized in Tracer v1.4 (Rambaut et al. 2018). The initial 25% of the trees were discarded as burn‐in. A consensus tree was then generated using the maximum‐clade‐credibility criterion and mean node weights with TreeAnnotator v2.7 (Helfrich et al. 2018) and visualized using FigTree v1.4 (Rambaut 2014).

Additionally, uncorrected p‐distances with 1000 bootstrap replicates were calculated for pairwise distances between the species of *Eurythenes* and the outgroups for the *16S* and *COI* genes separately, using MEGA X v11 (Kumar et al. 2018).

### 2.3 Demographic and Diversification Analyses

Changes in the effective population size (*Ne*) of *Eurythenes* were characterized by generating Bayesian skyline plots (BSP) using the Birth‐Death model with the concatenated alignment of the *16S* and *COI* genes in BEAST v2.7 (Bouckaert et al. 2014). The model was selected using bModelTest (Bouckaert and Drummond 2017). The MCMC chains were run for 5 × 10^8^ iterations, sampling every 1000 generations. The convergence of the analysis was checked using Tracer v1.7 (Rambaut et al. 2018), ensuring an ESS > 200 for all parameters.

Heterogeneity in the diversification rates was tested by applying the Bayesian Analysis of Macroevolutionary Mixtures (BAMM) package in R v.4.3 (Rabosky et al. 2014; RStudio Team Rs 2020). BAMM simulates posterior distributions of rate‐shift configurations using the reversible‐jump Markov Chain Monte Carlo (rjMCMC) method, accounting for rate variation over time and among lineages (Rabosky et al. 2014). The priors were obtained with BAMMtools, using the BI tree generated by BEAST v2.7 (Bouckaert et al. 2014). The evolutionary rate parameters were as follows: expected number of shifts = 1.0, lambdaInitPrior = 1.214, lambdaShiftPrior = 0.097, and muInitPrior = 1.214. The Poisson rate prior parameter of 1.0 was selected as a conservative approach.

The analysis was performed by running four independent MCMC chains for 1 × 10^7^ generations, saving every 1000 generations, and a burn‐in of 25%. The convergence of the chains was considered stable when the ESS > 200, using the *coda* package in R v.4.3 (Plummer et al. 2006). The rate‐shift configurations for the diversification analysis were retrieved with the highest posterior probability using the “getBestShiftConfiguration” function in BAMMtools. Plots of rates over time were generated for speciation (*λ*), extinction (*μ*), and diversification (*r*).

### 2.4 Ancestral Area Reconstruction Analyses

The ancestral range estimates (ARE) for the *Eurythenes* were performed using the *BioGeoBEARS* package in R v.4.3 (Matzke 2013). This approach implements various models, including the LaGrange dispersal‐extinction cladogenesis model (DEC, Ree and Smith 2008), a likelihood‐based version of DIVA (DIVALIKE), and a likelihood‐based version of BayArea (BayArea‐like). The addition of the founder effect as a free parameter (J) to each model was used. The J parameter considers that a descendant lineage immediately occupies a new area through long‐distance dispersal, which differs from each parental lineage. Ten biogeographic areas were user‐defined based on the distribution of *Eurythenes* taxa and their alignment to proposed deep‐ocean floor biogeographic regions (Watling et al. 2013): (1) Southwest Pacific Ocean, (2) Arctic Ocean, (3) Northwest Pacific Ocean, (4) Southern Ocean, (5) West Indian Ocean, (6) Southeast Pacific Ocean, (7) Central Pacific Ocean, (8) East Indian Ocean, (9) North Atlantic Ocean, and (10) South Atlantic Ocean. A maximum‐likelihood approximation tree was used to infer the probability of the ancestral area. The best‐fitting model was selected based on the AICw scores to perform biogeographic stochastic mapping (BSM) as part of the *BioGeoBEARS* package. The present‐day distributions were mapped to the tips of the BI phylogeny, and then BSM was used to infer ancestral states at internal nodes. 100 BSM iterations were conducted to estimate the frequencies and biogeographic events with the highest support values.

### 2.5 Drivers of Diversification

To evaluate whether changes in diversification rates could be due to palaeoceanographic factor such as ocean temperature, we used models implemented in the *RPANDA* package in R v.4.3 (Morlon et al. 2016), which allowed speciation and extinction rates to vary according to an environmental variable. We designed four separate paleoenvironment‐dependent models to detect the relationship between changes in diversification rates and the environment, as follows: (1) the speciation rate varies with the environment, and the extinction rate is zero (BEnvVar); (2) the speciation rate varies with the environment, and the extinction rate is constant (BEnvVarDcst); (3) the speciation rate is constant, and the extinction rate varies with the environment (BcstDEnvVar); and (4) the speciation and extinction rates vary with the environment (BEnvVarDEnvVar). Temperature data was inferred from average δ^18^O measurements included in the *RPANDA* package (Epstein et al. 1953; Zachos, Dickens, and Zeebe 2008; Condamine et al. 2018). The generated models were evaluated using the Akaike Information Criterion (AIC) to determine how well each model fit the sea temperature data. The ΔAIC represents the difference between the AIC of each model and the lowest observed AIC (Burnham and Anderson 2004). Models with a ΔAIC greater than 2 are considered significantly better than alternative models. Additionally, we report the AIC weight (AICw), which can be interpreted as the probability that a given model is the best among the set of candidate models included in the selection process.

## 3 Results

### 3.1 Phylogenetic Analyses and Known Regional Distributions

**FIGURE 1:**
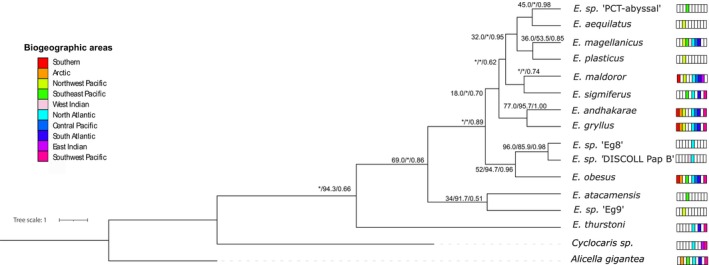
Bayesian phylogeny of *Eurythenes*, inferred from concatenated *16S* and *COI* in BEAST 2. The numbers on the nodes represent, from left to right, the Maximum Likelihood (ML), likelihood‐ratio test (SH‐aLRT), and Bayesian inference (BI) values. The presence of each species in the 10 biogeographic regions is indicated on the right by color. The asterisks (*) indicate differences between methodologies.

Global phylogenetic analysis of the genus *Eurythenes* produced consistent topologies between ML, SH‐aLRT and BI methodologies, identifying at least three distinct clades (Figure 1 and Figures S1–S3 in Data S2). The first clade, consisting solely of *E. thurstoni*, displayed moderate support values (ML = 99.0; SH‐aLRT = 94.3; BI = 0.66) and was distributed in the Pacific and Indian oceans. The second group comprised of *E. atacamensis* and *E*. sp. “*Eg9*” also demonstrated moderate support (ML = 34.0; SH‐aLRT = 91.7; BI = 0.51) and was found in the Pacific Ocean. The third group included *E. obesus* , *E*. sp. “*Eg8*,” and *E*. sp. “*DISCOLL Pap B*,” exhibited moderate to highsupport (ML = 52.0;SH‐aLRT = 94.7; BI = 0.96). Within this group, *E. obesus* showed a global distribution, whereas the other two species were restricted to the North Atlantic and/or Western Indian oceans. A fourth clade was observed both ML and BI approaches, with low to moderate support (ML = 22.0; BI = 0.70). This clade included the remaining eight species, which showed high variability in geographic distribution, ranging from region‐specific species (e.g., *E*. sp. “PCT‐abyssal”) to nearly cosmopolitan species (e.g., *E. maldoror*).

The largest genetic distances (*D*) were observed between *E. thurstoni* and *E*. sp. “*PCT‐abyssal*” and *E. gryllus* for the *16S* gene (*D* = 0.171), as well as between *E. atacamensis* and *E. obesus* for the *COI* gene (*D* = 0.171) (see Table S2 Data S2). In contrast, the smallest genetic distances were exhibited between *E. magellanicus* and *E. andhakarae* for the *16S* gene (*D* = 0.009), and between *E. magellanicus* and *E. plasticus* for the *COI* gene (*D* = 0.077).

### 3.2 Divergence Times

**FIGURE 2:**
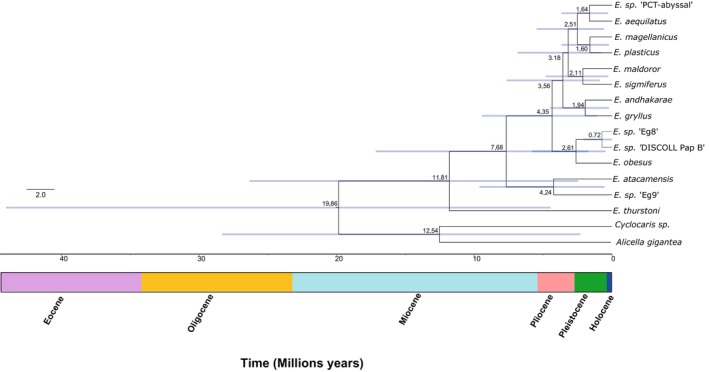
Time‐calibrated phylogeny of *Eurythenes* estimated from *16S* and *COI* in BEAST 2. The timescale was calibrated with the biogeographic scenario for the origin and diversification of *Eurythenes*. Node labels indicate the mean age, while blue bars represent the 95% highest posterior density interval (HPD). The mean divergence times are expressed in millions of years.

Bayesian phylogenetic reconstruction revealed the existence of three ancestral lineages that contributed to the diversification of the genus *Eurythenes* (Figure 2). The stem age of the clade is 19.86 Ma, and the initial branching event (or crown age) occurred ~11.81 Ma, leading to the differentiation of *E. thurstoni*. Subsequently, around the late Miocene (~7.68 Ma). Most current species diverged during the Early Pleistocene (~4.5–0.79 Ma).

### 3.3 Historical Demography

**FIGURE 3:**
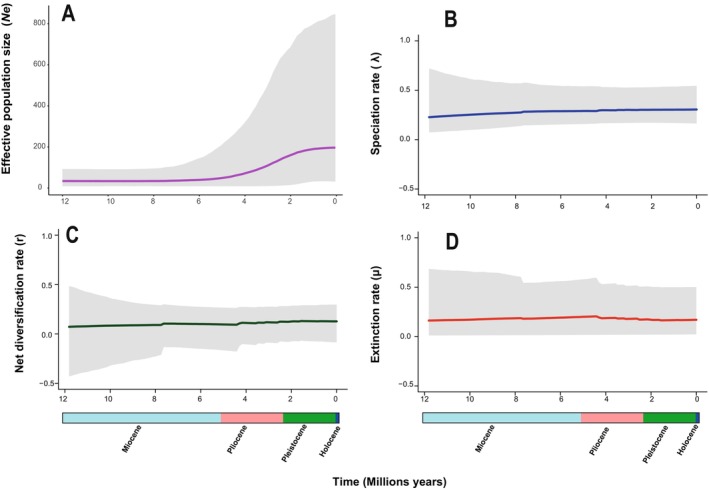
Bayesian skyline plots of *Eurythenes* showing changes in population size (A), speciation rate (B), net diversification (C), and extinction rate (D). Analyses were performed by concatenating the *16S* and *COI* genes. The continuous gray lines indicate the average values, while the light blue shading denotes the 95% credibility interval.

Reconstruction of the effective population size (*Ne*) of *Eurythenes* revealed a constant size of 33 individuals from the middle Miocene (~12 Ma) until the early Pliocene (~5.33 Ma) (Figure 3A). The relative population size showed small increase over timed, reaching an average of approximately 200 individuals in the Early Pleistocene (~1.8 Ma) and remaining constant until the Holocene (< 11,000 years).

Speciation rates (*λ*) and net diversification (r) for all *Eurythenes* species from the middle Miocene (~12 Ma) to the present remained almost constant, with a slight upward trend (*λ* = 0.26–0.31 species/Ma; *r* = 0.11–0.16) (Figure 3B,C). However, the extinction rate (*μ*) also showed general stability, with a slight decline since the Pliocene (~4 Ma) (*μ* = 0.19–0.16 y species/Ma) (Figure 3D).

### 3.4 Reconstruction of Ancestral Range

**FIGURE 4:**
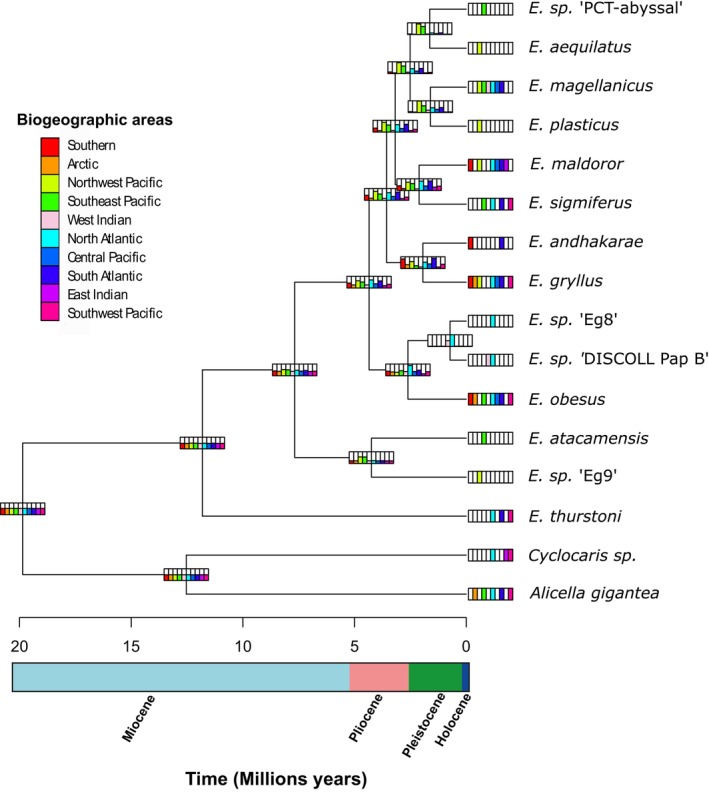
Ancestral range reconstruction of *Eurythenes* using BioGeoBEARS. The BAYAREALIKE model emerged as the best fit. Colored rectangles at the nodes indicate the inferred ancestral areas(s).

The ARE for *Eurythenes*, spanning from the middle Miocene (~11.81 Ma) to the present, revealed that the most ancient common ancestor had a global distribution, giving rise to both widely distributed and more restricted species (Figure 4). The best‐fitted model was BAYAREALIKE without the jump parameter (*J*) (Tables 1 and 2). The model‐derived parameters included the anagenetic dispersal rate (*d* = 0.053) and extinction rate (*e* = 0.29). BSM showed that 88.06% of speciation events were attributed to dispersal, while 13.93% were sympatric events (Table 3).

**TABLE 1:** Comparison of biogeographic models with and without the founder effect (J) implemented in BioGeoBEARS.

Models	LnL	AIC	AICw
DEC	−92.74	190.40	0.12
DEC+J	−92.54	193.10	0.03
DIVALIKE	−93.67	192.30	0.05
DIVALIKE+J	−93.52	195.00	0.01
BAYAREALIKE	−91.00	186.90	0.66
BAYAREALIKE+J	−91.01	190.00	0.14

*Note:* For each model, the likelihood (LnL), Akaike Information Criterion (AIC), and AIC weight (AICw) are indicated.

**TABLE 2:** Comparison of biogeographic models with and without the founder effect (J) implemented in BioGeoBEARS. The likelihood values for the alternative (LnL alt) and null (LnL null) models are displayed, along with the statistic D and its significance level (*p*‐value).

Models	Null model	LnL alt	LnL null	*D*	*p*
DEC+J	DEC	−92.54	−92.74	0.400	0.53
DIVALIKE+J	DIVALIKE	−93.52	−93.67	0.310	0.58
BAYAREALIKE+J	BAYAREALIKE	−91.01	−91.00	−0.003	1.00

*Note:* The likelihood values for the alternative (LnL alt) and null (LnL null) models are displayed, along with the statistic *D* and its significance level (*p*‐value).

**TABLE 3:** Results of the biogeographic stochastic mapping (BSM) for *Eurythenes* according to the BAYAREALIKE model implemented in BioGeoBEARS.

Speciation mode	Type	Mean	SD	Percent
Dispersal	Founder event	0	0	0
	Range expansion	110.7	8.54	88.06
Vicariance	Vicariance	0	0	0
Within‐area	Speciation	15	0	13.93
Total		125.7	8.54	100

*Note:* Mean values, standard deviations (SD), and percentages of each speciation mode are shown.

The ARE of *Eurythenes* was situated in ten biogeographical regions with equal probability, and it was during this time that the speciation of *E. thurstoni* occurred. Subsequently, in the late Miocene (~7.68 Ma), the ancestral area of other congeners was redistributed across all biogeographical regions. However, in the early Pliocene (~4.24 Ma), the ARE of the common ancestor of *E. atacamensis* and *E*. sp. “Eg9” was more likely located in the Pacific Ocean. Almost simultaneously (~4.24 Ma), the ERA of the remaining species showed a higher probability in the Pacific and Atlantic Oceans, giving rise to two groups during the Late Pleistocene (~3.46–2.61 Ma). One of these groups, the common ancestor of *E. obesus* , *E*. sp. “DISCOLL Pap B,” and *E*. sp. “Eg8,” had its ERA restricted to the North Atlantic and subdivided into three subgroups during the Early Pleistocene (~2.51–1.94 Ma). The first subgroup had its ARE mainly in the Pacific, being the common ancestor of *E*. sp. “PCT‐abyssal,” *E. aequilatus, E. magellanicus*, and *E. plasticus*. The second subgroup had an ARE restricted to the Pacific and Atlantic Oceans, giving rise to a clade of *E. maldoror* and *E. sigmiferus*. The third subgroup had an ARE mainly in the South Atlantic and Southern Ocean, giving rise to the common ancestor of *E. andhakarae* and *E. gryllus* .

### 3.5 Paleoceanographic Variation and Diversification Models

**FIGURE 5:**
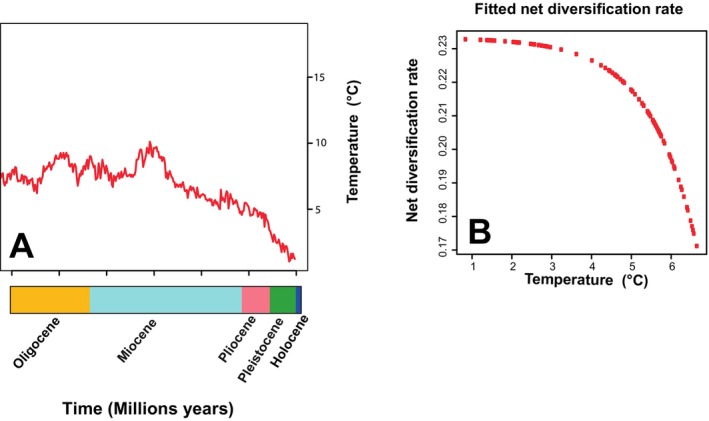
Changes in temperature from the Oligocene to the present (A). The relationship between the net diversification rate and temperature is modeled by BcstDEnvVar, where the speciation rate is constant, and the extinction rate varies with the environment (B).

Paleoceanographic data revealed variations in ocean temperature over the last 30 Ma (Figure 5A). When evaluating the fit of diversification models based on ocean temperature, analyses showed that both the BEnvVar model (where speciation rate varies with environmental conditions and extinction rate is zero) and the BcstDEnvVar model (where speciation rate remains constant and extinction rate varies with the environment) displayed the best fits, with the highest AICw values (> 0.41). However, evaluating the ΔAIC for both models revealed a value < 2, indicating little evidence of significant differences in their ability to explain the effects of ocean temperature variability on the diversification of *Eurythenes* (ΔAICc < 2; Table 4). Nonetheless, consistent with the results obtained through the BAMM approach, the BcstDEnvVar model was selected as the best fit. This model, which incorporates extinction rate, showed an inverse relationship between changes in temperature and the net diversification rate. Specifically, it was observed that as sea temperature increases, the diversification rate decreases (Figure 5B).

**TABLE 4:** Results diversification analyses for *Eurythenes*, adjusting for paleoceanographic variables.

Models	Variable	LnL	AICc	AICw	ΔAIC
BEnvVar	Temperature	−31.18	67.46	0.48	0.00
BEnvVarDcst	Temperature	−31.18	70.77	0.09	3.21
BcstDEnvVar	Temperature	−29.68	67.78	0.41	0.29
BEnvVarDEnvVar	Temperature	−31.18	74.82	0.01	7.35

*Note:* The evaluated models were: (1) speciation rate varies with the environment, and the extinction rate is zero (BEnvVar); (2) speciation rate varies with the environment, and the extinction rate is constant (BEnvVarDcst); (3) speciation rate is constant, and the extinction rate varies with the environment (BcstDEnvVar); and (4) both speciation and extinction rates vary with the environment (BEnvVarDEnvVar). For each model, the likelihood (LnL), corrected Akaike Information Criterion (AIC), AIC weight (AICw), and delta AIC (ΔAIC) are indicated.

## 4 Discussion

*Eurythenes*, a genus with a global distribution, offers a unique perspective for studying species diversification and radiation in deep‐sea environments. Our analyses of its evolutionary history suggest that oceanographic processes, such as fluctuations in sea level and temperature, have significantly affected its speciation dynamics. We propose that the origin of this genus dates to the middle Miocene (~11.81 Ma), coinciding with a reorganization of the lithosphere that triggered changes in deep circulation and oceanographic conditions (e.g., McClain and Hardy 2010; Herbert et al. 2016). This large‐scale geological event may have been the primary driver behind the emergence of at least 14 distinct species over the past few million years (Havermans 2016). On the other hand, paleoceanographic changes have likely had a significant impact on the evolutionary history of *Eurythenes* in the deep ocean due to their high sensitivity to environmental variations stemming from their size, mobility, and dependence on environmental conditions. For instance, major geological events, such as the drift of Africa and Arabia leading to the closure of the Tethys Ocean, along with the collision of the Australian plate during the early‐middle Miocene (20–15 Ma) (Kennett 1977; Kennett, Elmstrom, and Penrose 1985), resulted in a change in deep ocean circulation. This transition from a predominantly global equatorial circulation to a more restricted meridional circulation likely affected the distribution of the common ancestor of *Eurythenes* (Kennett, Elmstrom, and Penrose 1985; Grigg 1988).

Our results suggest that the common ancestor of *Eurythenes* had a global distribution, and that the predominant meridional circulation pattern may have influenced its dispersal during the Miocene. Furthermore, during the same period, the mechanism of deep‐water generation shifted from halothermal to thermohaline circulation (McClain and Barry 2010; Copilaş‐Ciocianu, Borko, and Fišer 2020). Under halothermal circulation, deep waters originated in the Tethys Sea, creating high‐temperature, low‐oxygen conditions on the seafloor. In contrast, thermohaline circulation, originating in high latitudes, resulted in a cold, well‐oxygenated seafloor (Jażdżewska et al. 2021). This shift in circulation patterns may have facilitated the colonization of vast areas of the seafloor by *Eurythenes*, which were adapted to low‐temperature, high‐oxygen conditions (Copilaş‐Ciocianu, Borko, and Fišer 2020).

In the last 4 Ma, an increase in the net diversification rate and the effective population size of *Eurythenes* has been observed. This phenomenon may be related to geological events, such as the closure of the Isthmus of Panama between the middle Miocene and the Pliocene (15–3 Ma), which intensified deep circulation, allowing for the dispersal and speciation of *Eurythenes* in a favorable environment (Bacon et al. 2015; Lessios 2015; Marko, Eytan, and Knowlton 2015). In the same context, it is relevant to mention that our model estimate of the effective population size of *Eurythenes* is relatively low, at about 200 individuals on average. While the small sample size may drive this low historical Ne and not based on genome‐wide analyses of homozygosity, studies on other marine and terrestrial arthropods have indicated that their effective population sizes rarely exceed 1000 individuals (Xun et al. 2016; Hupało et al. 2019). Further, deep‐water bivalves are shown to have smaller effective population sizes than shallow ones (Jennings, Etter, and Ficarra 2013). This rather small effective population size could result from the influence of factors such as inbreeding, complex life cycles, ontogenetic migration, parental care, and low dispersal capacity, all of which may be relevant to *Eurythenes* (d'Udekem d'Acoz and Havermans 2015; Weston et al. 2021; Jamieson and Weston 2023).

Glacial periods are another explanatory factor that may have shaped the evolutionary history and current distribution of *Eurythenes*. The global increase in ice volume, the consequent drop in sea levels, the decrease in temperatures, and the increase in productivity likely influenced population dynamics and the diversification of *Eurythenes*. These factors would have promoted speciation and adaptation to new ecological niches in various oceanic regions (Clark et al. 2006; Elderfield et al. 2012; McClymont et al. 2013; Kender et al. 2016). Furthermore, the deep ocean, which remained more stable than the surface layers, may have acted as a refuge, facilitating specialization through efficient species adaptation to exploit available resources (McClain and Hardy 2010).

By tracing the ancestral origins of *Eurythenes* in the global ocean, we have identified a complex interaction of dispersal processes that has shaped its evolution. Due to the wide bathymetric range, these phenomena are not mutually exclusive and may have interacted with environmental factors to promote diversification. Amphipods can easily disperse, and several species of *Eurythenes* have distributions that span trenches and abyssal plains. This fact, along with the thermohaline current and its recirculation time (1500 years), may have facilitated the colonization of new areas and the differentiation of its congeners through geographic isolation or natural selection. However, it has been observed that dispersal and speciation rates can be correlated in some organisms (O'Donovan, Meade, and Venditti 2018; Avaria‐Llautureo et al. 2021), and highly dispersive lineages, such as *Eurythenes*, may be less prone to extinction or more capable of speciating through geographic expansion across barriers in the ocean (Dueñas et al. 2016).

Regarding the type of speciation processes driving the *Eurythenes* diversification, we could refer to some deep‐sea crustaceans experiencing sympatric speciation, as observed in isopods and peracarids (Raupach et al. 2007), and in *Eurythenes* from our results. This speciation process is usually associated with divergent natural selection, which prevails over the homogenizing effects of gene flow (Bowen et al. 2013), manifesting through direct selection for reproductive isolation, affecting both temporal and behavioral isolation (Jones et al. 2003; Rocha et al. 2005; Crow, Munehara, and Bernardi 2010). Since *Eurythenes* are predominately scavengers, the observed sympatry could be due to the occupation of different ecological niches and specializations through predation, feeding on marine detritus, or positioning in the water column. This process has likely allowed its differentiation and adaptation to distinct ocean basins and even more localized regions, such as trenches (Jamieson and Weston 2023).

Ultimately, interpreting studies on speciation, dating, and determining ancestral areas should be cautiously approached because calibrating a molecular clock can lead to erroneous conclusions (Pulquerio and Nichols 2007). For example, the absence of a fossil record or the dating of a specific geological event for the speciation of *Eurythenes* limits the accuracy of molecular clock calibration. It complicates inferences about speciation and extinction rates. However, despite these limitations, substitution rates in molecular clock calibration have produced results similar to those obtained using other methods (Bousfield 1983; Copilaş‐Ciocianu and Petrusek 2015). Our analyses are also limited by incomplete bathymetric and geographic sampling, particularly in the Southeast Pacific Gyre and the Mid‐Atlantic Ridge. This could be attributed to the largely unexplored deep‐sea ecosystem inhabited by this group, which hinders sample acquisition and highlights the need for more integrative taxonomy. Another influential factor could be the presence of undescribed species, which could lead to inaccurate phylogenetic reconstructions and introduce biases in dating and diversification analyses, especially in species exhibiting cryptic speciation, as is the case in the genus *Eurythenes* (Chang, Rabosky, and Alfaro 2020). Nevertheless, our time‐calibrated phylogeny of *Eurythenes*, which includes all species identified to date and encodes those yet to be identified, represents a crucial first step in understanding the evolution and factors that may have shaped it, leading this genus to colonize the most inhospitable environments of the ocean.

## Author Contributions

**Carolina E. González:** conceptualization (lead), formal analysis (lead), investigation (lead), methodology (lead), writing – original draft (lead), writing – review and editing (lead). **Johanna N. J. Weston:** conceptualization (lead), investigation (lead), methodology (lead), writing – original draft (lead), writing – review and editing (lead). **Reinaldo Rivera:** conceptualization (equal), methodology (equal), writing – original draft (equal), writing – review and editing (equal). **Marcelo Oliva:** investigation (equal), writing – original draft (equal). **Rubén Escribano:** funding acquisition (lead), writing – original draft (equal), writing – review and editing (equal). **Osvaldo Ulloa:** funding acquisition (lead), supervision (equal), writing – review and editing (equal).

## Conflicts of Interest

The authors declare no conflicts of interest.

## Supporting information


Data S1.

Data S2.

## Data Availability

The dataset, along with the associated scripts required to replicate all, is available on Dryad. https://datadryad.org/stash/share/m8ZmxParOFBJtijDZwPK0olzZkhJXzariWTvTxPq5WI.
