# Supplementary material for: Biogeographic Insights Into the Late Miocene Diversification of the Giant Deep‐Ocean Amphipod Eurythenes
Source: Ecol Evol. 2025 Jan 23;15(1):e70730. doi: 10.1002/ece3.70730 (PMC11756929; doi:10.1002/ece3.70730)
Supplement: Supplementary file 2 — Data S2. [file ECE3-15-e70730-s002.docx]

**Supplementary Information**

**Table S1.** For each species used in this analysis, the GenBank codes for 16S rRNA and COI, bibliographic reference, phylogenetic position in the calibrated tree (Group), latitude (Lat), longitude (Long), sampling depth, sampling location, and biogeographic province are provided.

| **Species** | **16S rRNA Genbank** | **COI Genbank** | **Reference** | **Group** | **Lat.** | **Long.** | **Depth (m)** | **Locality** | **Biogeographic Areas** |
| --- | --- | --- | --- | --- | --- | --- | --- | --- | --- |
| *Eurythenes obesus* | KP456144 | KP713954 | Ritchie, Jamieson and Piertney, 2015 | In group | -6.58 | -81.52 | 915 | Peru-Chile Trench | Southeast Pacific |
| *Eurythenes thurstoni* | U40449 | No data | France and Kocher, 1996 | In group | 25.00 | -78.00 | 1122 | NW Channel, Bahamas | North Atlantic |
| *Eurythenes gryllus* | JX887060 | JX887132 | Havermans *et al.*, 2013 | In group | 79.82 | 5.48 | 2464 | Eastern Fram Strait | Arctic |
| *Eurythenes sp. 'DISCOLL Pap B '* | MZ197473 | MZ197342 | Kniesz *et al.*, 2022 | In group | -27.65 | 73.89 | 2508 | Central Indian Ridge | West Indian |
| *Eurythenes sp. 'Eg8'* | U40439 | No data | France and Kocher, 1996 | In group | 59.00 | 21.00 | 2900 | Iceland Basin | North Atlantic |
| *Eurythenes sp. 'Eg9'* | U40446 | No data | France and Kocher, 1996 | In group | 20.00 | 169.00 | 3193 | Horizon Guyot slope | Northwest Pacific |
| *Eurythenes magellanicus* | JX887073 | JX887145 | Havermans *et al.*, 2013 | In group | -26.55 | -35.18 | 4480 | Brazil Basin | South Atlantic |
| *Eurythenes sp. 'PCT-abyssal'* | KP456140 | KP713957 | Ritchie, Jamieson and Piertney, 2015 | In group | -6.20 | -81.67 | 4602 | Peru-Chile Trench | Southeast Pacific |
| *Eurythenes maldoror* | MN251321 | MN262171 | Weston *et al.*, 2021 | In group | -3.16 | 82.43 | 4757 | Afanasy Nikitin Seamount | East Indian |
| *Eurythenes sigmiferus* | ON390987 | ON385094 | Weston *et al.*, 2023 | In group | -42.77 | 10.05 | 5493 | Agulhus Fracture Zone | South Atlantic |
| *Eurythenes andhakarae* | ON390983 | ON385095 | Weston *et al.*, 2023 | In group | -42.77 | 10.05 | 5493 | Agulhus Fracture Zone | South Atlantic |
| *Eurythenes plasticus* | MT021437 | MT038070 | Weston *et al.*, 2020 | In group | 12.59 | 144.85 | 6010 | Mariana Trench | Northwest Pacific |
| *Alicella gigantea* | KP456083 | KP713893 | Ritchie, Jamieson and Piertney, 2015 | Outgroup | -32.55 | -117.23 | 7000 | Kermadec Trench | Southwest Pacific |
| *Eurythenes aequilatus* | LC229090 | LC229094 | Narahara-Nakano, Nakano and Tomikawa, 2018 | In group | 44.58 | 144.70 | 1582 | Sea of Okhotsk | Northwest Pacific |
| *Eurythenes atacamensis* | PP453557 | PP430572 | This studio | In group | -23.57 | -71.46 | 6500 | Peru-Chile Trench | Southeast Pacific |
| *Cyclocaris sp.* | KF430272 | No data | Corrigan *et al.*, 2014 | Outgroup | -6.50 | -11.15 | 1975 | Mid-Atlantic ridge | South Atlantic |
| *Cyclocaris sp.* | No data | KP713899 | Ritchie, Jamieson and Piertney, 2015 | Outgroup | -24.96 | 171.05 | 4100 | Tonga Trench | Southwest Pacific |

**Table S2**. Genetic distance among *Eurythenes* species based on *16S* and *COI* genes. Distances for *16S* are reported below the diagonal, and *COI* values are presented above. The first column displays the names of each group or species identified by the numbers. The "NA" indicate the impossibility of calculation due to the lack of sequence for a specific gene.

__________________________________________________________________________________________________________________

1 2 3 4 5 6 7 8 9 10 11 12 13 14

__________________________________________________________________________________________________________________

1. *E. aequilatus* - 0.130 0.157 NA 0.123 NA 0.123 0.102 0.105 0.166 0.104 0.110 0.123 NA

2. *E. andhakarae* 0.028 - 0.148 NA 0.140 NA 0.086 0.095 0.100 0.163 0.123 0.127 0.111 NA

3. *E. atacamensis* 0.114 0.073 *-* NA 0.152 NA 0.151 0.152 0.149 0.171 0.158 0.163 0.158 NA

4. *E*. sp. ‘Eg8’ 0.060 0.024 0.105 - NA NA NA NA NA NA NA NA NA NA

5. *E. sp.* ‘Pap B’ 0.056 0.029 0.118 0.005 - NA 0.127 0.123 0.124 0.122 0.117 0.139 0.132 NA

6. *E*. sp. ‘Eg9’ 0.107 0.057 0.099 0.088 0.100 - NA NA NA NA NA NA NA NA

7. *E. gryllus* 0.058 0.023 0.110 0.045 0.051 0.099 - 0.103 0.098 0.159 0.113 0.120 0.110 NA

8. *E. magellanicus* 0.048 0.009 0.093 0.045 0.057 0.083 0.044 - 0.090 0.154 0.104 0.077 0.096 NA

9. *E. maldoror* 0.051 0.028 0.085 0.048 0.050 0.084 0.030 0.034 - 0.139 0.113 0.125 0.101 NA

10. *E. obesus* 0.093 0.055 0.110 0.059 0.077 0.121 0.072 0.068 0.065 *-* 0.169 0.165 0.151 NA

11. *E*. sp. ‘PCT-abyssal’ 0.055 0.050 0.119 0.063 0.061 0.113 0.068 0.056 0.065 0.097 - 0.106 0.130 NA

12. *E. plasticus* 0.038 0.023 0.086 0.053 0.064 0.076 0.043 0.026 0.038 0.071 0.056 - 0.104 NA

13. *E. sigmiferus* 0.061 0.041 0.071 0.047 0.059 0.084 0.046 0.041 0.031 0.082 0.075 0.046 *-* NA

14. *E. thurstoni* 0.167 0.159 0.178 0.159 0.166 0.169 0.171 0.165 0.161 0.162 0.171 0.164 0.182 -

_______________________________________________________________________________________________________________

**Figure S1.** Maximum Likelihood phylogeny of *Eurythenes*, inferred from concatenated *COI* and *16S* genes. The numbers on the nodes represent the posterior Maximum Likelihood values.


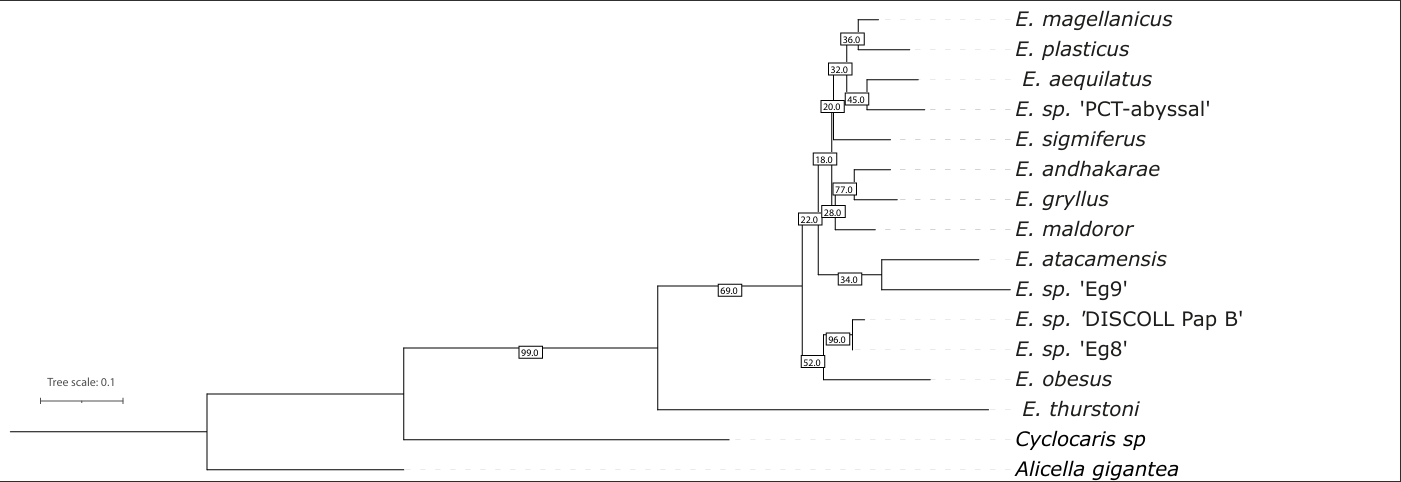


**Figure S2**. Likelihood-ratio test phylogeny of *Eurythenes,* inferred from concatenated *COI* and *16S* genes. The numbers on the nodes represent the posterior likelihood-ratio values.


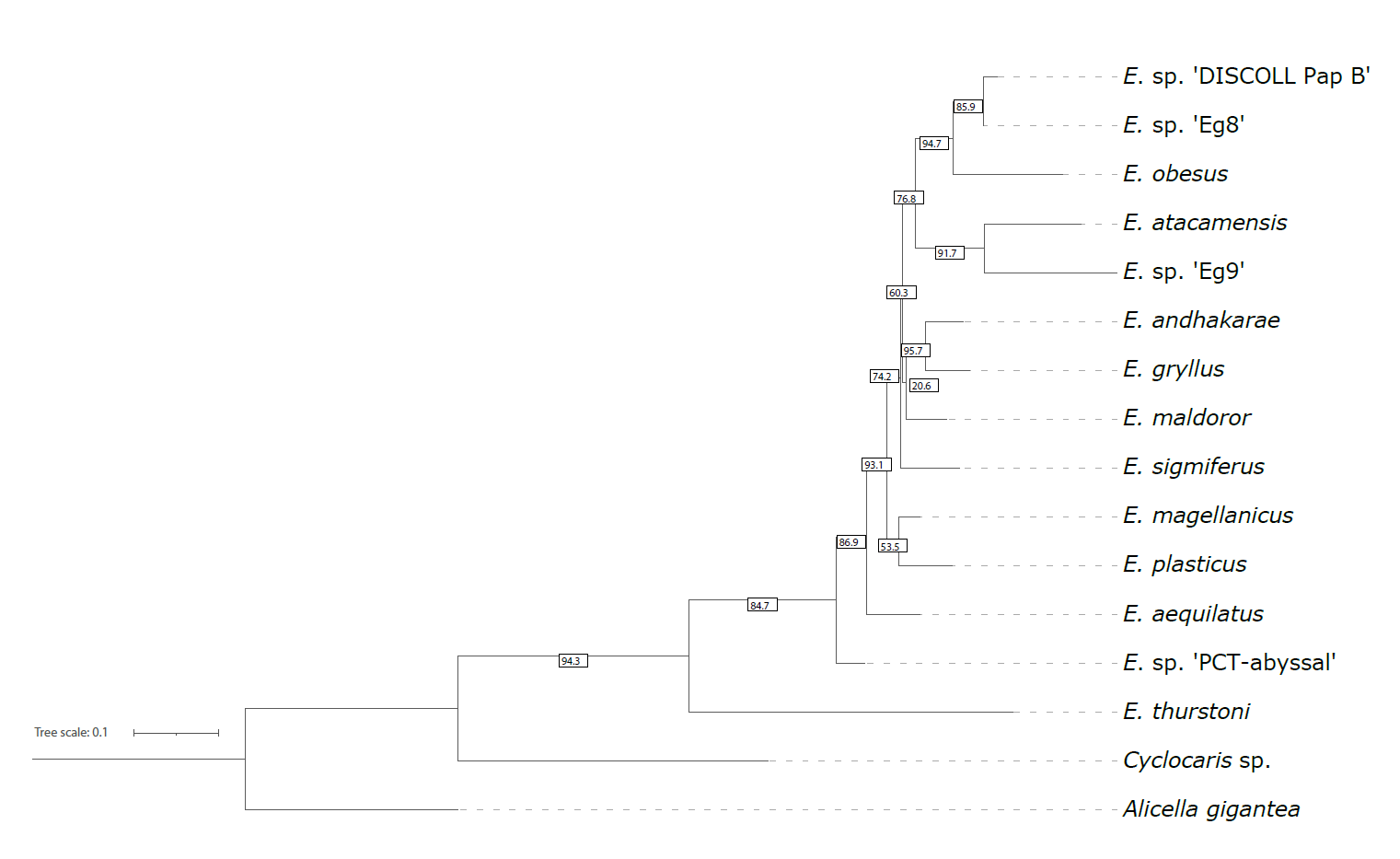


**Figure S3**. Bayesian phylogeny of *Eurythenes* inferred from concatenated *COI* and *16S* genes. The numbers on the nodes represent the Bayesian inference values.


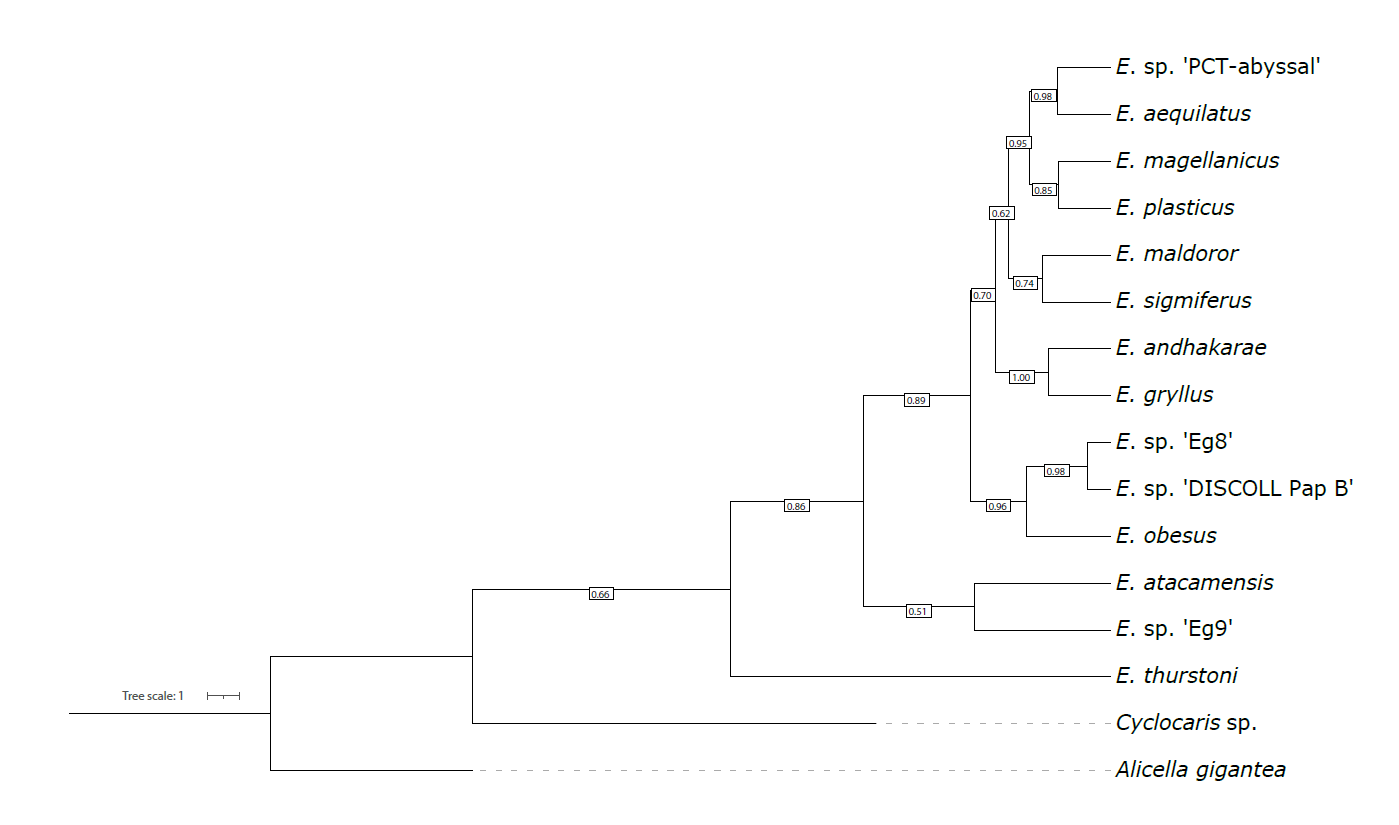


**References**

Corrigan, L. J. *et al.* (2014) ‘Adaptive Evolution of Deep-Sea Amphipods from the Superfamily Lysiassanoidea in the North Atlantic’, *Evolutionary Biology*, 41(1), pp. 154–165. doi: 10.1007/S11692-013-9255-2/FIGURES/5.

France, S. C. and Kocher, T. D. (1996) ‘Geographic and bathymetric patterns of mitochondrial 16S rRNA sequence divergence among deep-sea amphipods, Eurythenes gryllus’, *Marine Biology*, 126(4), pp. 633–643. doi: 10.1007/BF00351330.

Havermans, C. *et al.* (2013) ‘Genetic and Morphological Divergences in the Cosmopolitan Deep-Sea Amphipod Eurythenes gryllus Reveal a Diverse Abyss and a Bipolar Species’, *PLoS ONE*, 8(9). doi: 10.1371/journal.pone.0074218.

Kniesz, K. *et al.* (2022) ‘DNA Barcoding of Scavenging Amphipod Communities at Active and Inactive Hydrothermal Vents in the Indian Ocean’, *Frontiers in Marine Science*, 8, p. 752360. doi: 10.3389/FMARS.2021.752360/BIBTEX.

Narahara-Nakano, Y., Nakano, T. and Tomikawa, K. (2018) ‘Deep-sea amphipod genus Eurythenes from Japan, with a description of a new Eurythenes species from off Hokkaido (Crustacea: Amphipoda: Lysianassoidea)’, *Marine Biodiversity*, 48(1), pp. 603–620. doi: 10.1007/s12526-017-0758-4.

Ritchie, H., Jamieson, A. J. and Piertney, S. B. (2015) ‘Phylogenetic relationships among hadal amphipods of the Superfamily Lysianassoidea: Implications for taxonomy and biogeography’, *Deep-Sea Research Part I: Oceanographic Research Papers*, 105, pp. 119–131. doi: 10.1016/j.dsr.2015.08.014.

Weston, J. N. J. *et al.* (2020) ‘New species of Eurythenes from hadal depths of the Mariana Trench, Pacific Ocean (Crustacea: Amphipoda)’, *Zootaxa*, 4748(1), pp. 163–181. doi: 10.11646/zootaxa.4748.1.9.

Weston, J. N. J. *et al.* (2021) ‘Scavenging amphipods from the Wallaby-Zenith Fracture Zone: Extending the hadal paradigm beyond subduction trenches’, *Marine Biology*, 168(1), pp. 1–14. doi: 10.1007/S00227-020-03798-4/FIGURES/4.

Weston, J. N. J. *et al.* (2023) ‘Eurythenes sigmiferus and Eurythenes andhakarae (Crustacea: Amphipoda) are sympatric at the abyssal Agulhas Fracture Zone, South Atlantic Ocean, and notes on their distributions’, *Deep Sea Research Part I: Oceanographic Research Papers*, 196, p. 104050. doi: 10.1016/J.DSR.2023.104050.
